# Relationship between body mass index and mean arterial pressure in normotensive and chronic hypertensive pregnant women: a prospective, longitudinal study

**DOI:** 10.1186/s12884-015-0711-0

**Published:** 2015-10-30

**Authors:** Luís Guedes-Martins, Mariana Carvalho, Catarina Silva, Ana Cunha, Joaquim Saraiva, Filipe Macedo, Henrique Almeida, A. Rita Gaio

**Affiliations:** Department of Women and Reproductive Medicine, Hospital Centre of Porto EPE, Largo Prof. Abel Salazar, 4099-001 Porto, Portugal; Department of Experimental Biology, Faculty of Medicine, University of Porto, 4200-319 Porto, Portugal; Institute of Biomedical Sciences Abel Salazar, University of Porto, 4050-313 Porto, Portugal; Department of Mathematics, Faculty of Sciences, University of Porto, 4169-007 Porto, Portugal; Department of Cardiology, Faculty of Medicine, University of Porto, 4200-319 Porto, Portugal; Obstetrics-Gynaecology, CUF-Hospital Porto, 4100 180 Porto, Portugal; CMUP-Centre of Mathematics, University of Porto, 4169-007 Porto, Portugal

**Keywords:** Pregnancy, Hypertension, Body mass index, Mean arterial pressure

## Abstract

**Background:**

Being overweight is associated with both higher systolic blood pressure (SBP) and diastolic blood pressure (DBP) during pregnancy and increased risk of gestational hypertensive disorders. The objective of this study was to determine and quantify the effect of body mass index (BMI) on mean arterial pressure (MAP) at several time points throughout pregnancy in normotensive (NT) and chronic hypertensive pregnant (HT) women.

**Methods:**

A prospective longitudinal study was carried out in 461 singleton pregnancies (429 low-risk and 32 with chronic arterial hypertension), with measurements taken at the 1^st^, 2^nd^, and 3^rd^ trimesters and at delivery. Linear mixed-effects regression models were used to evaluate the time-progression of BMI, SBP, DBP and MAP during pregnancy (NT vs. HT). The longitudinal effect of BMI on MAP, adjusted for the hypertensive status, was investigated by the same methodology.

**Results:**

BMI consistently increased with time in both NT and HT women. In contrast, MAP decreased during the first half of pregnancy, after which it increased until the moment of delivery in both groups. A 5-unit increase in BMI was predicted to produce an increase of approximately 1 mmHg in population MAP values. This effect is independent from the time period and from hypertensive status.

**Conclusions:**

In both NT and HT pregnant women, MAP is strongly (and significantly) influenced by increases in BMI.

## Background

Chronic arterial hypertension is a serious disorder; if left untreated, it can lead to serious health outcomes, mostly affecting target organs such as the heart, brain, kidney and retina [1].

Women diagnosed with hypertension who become pregnant are at an increased risk for several pregnancy complications, including superimposed preeclampsia [2, 3], foetal growth restriction [4], preterm delivery [5], placental abruption [6], and caesarean section [3, 5]. In addition, because chronic arterial hypertension affects 3–5 % of pregnancies [7, 8], it alone is a matter of concern and is increasingly encountered [3]. Obesity is the main risk factor contributing to this increased prevalence; its frequency is increasing among pregnant women, and it is a well-known risk factor for both adverse maternal [9] and neonatal outcomes [10, 11]. In fact, increased adiposity during normal pregnancy has been consistently associated with the same medical complications that are associated with chronic arterial hypertension in pregnant women (as described above) [12]. However, the mechanisms for these associations are not completely understood.

Several studies have examined the effects of maternal weight on blood pressure levels during different periods of normal pregnancy [10, 13–16]. The results suggest that overweight, obesity and morbid obesity are associated with higher systolic (SBP) and diastolic blood pressure (DBP) during pregnancy and increased risks of gestational hypertensive disorders [9].

Nevertheless, studies that have effectively quantified the effects of weight gain on maternal blood pressure during pregnancy are lacking. It has been reasoned that additional data on the effects of increased body mass index (BMI) on mean arterial pressure (MAP) during normal pregnancy would be provided by a parallel study in women with long-term stable essential hypertension, which is a prevalent condition and a known risk factor for serious gestational disorders [5, 7, 17].

Based on these considerations, this study aimed to determine and quantify the effects of BMI on MAP at several time points throughout pregnancy, in normotensive (NT) and chronic hypertensive (HT) pregnant women.

## Methods

### Ethics

The research protocol was approved by the local ethics committee (IRB protocol number: 133/10 [086-DEFI/126-CES]) of the Centro Hospitalar do Porto – Unidade Maternidade Júlio Dinis (CHP-MJD), and all of the subjects provided written consent upon receiving an adequate explanation of the study. The methods were carried out in accordance with the approved protocol.

### Study population and design

Between January 2010 and December 2012 a total of 578 pregnant Caucasian women were recruited to participate in the study. According to local pregnancy health policies, the women were referred by their family doctors to the CHP-MJD.

During their first appointment the women were observed by a senior specialist who reviewed their medical history, verified the absence of diabetes and other endocrine disorders, immune diseases, renal diseases, structural heart diseases, haematological conditions and chronic infections; gestational age (GA) was also checked by ultrasonography between 11 and 14 weeks [18].

The inclusion criteria were as follows: (1) singleton pregnancy and gestational age ≤ 14 weeks, and (2) healthy status or stable chronic arterial hypertension without known target organ involvement.

The exclusion criteria were as follows: (1) patients with multiple gestations, coagulopathy, haematological pathology, diabetes, or any pregnancy-induced hypertension including preeclampsia, and (2) patients who refused to participate. Subjects were also excluded from the study if they had a preterm delivery (birth < 37^th^ gestational week), were lost to follow-up, needed antihypertensive medication, or experienced foetal death.

Chronic arterial hypertension was defined as a blood pressure of 140/90 mmHg on more than two occasions before 20 weeks of gestation or after 20 weeks of pregnancy if it persisted beyond 12 weeks postpartum [17].

Before pregnancy, the majority of the hypertensive patients required multiple medications, including thiazide, angiotensin converting enzyme inhibitor, angiotensin receptor blockers, or calcium channel blockers, to control their hypertension. After their first appointment, antihypertensive drugs were discontinued, and their blood pressure was closely monitored. Antihypertensive therapy was restarted if a patient experienced a persistent diastolic pressure between 95 and 99 mmHg or if a systolic pressure ≥ 150 mmHg was observed at any time during their pregnancy. For women with chronic hypertension who enter pregnancy not on antihypertensive treatment, the antihypertensive treatment was initiated when blood pressures were consistently > 160 mmHg systolic and/or > 105 mmHg diastolic [17].

### Definition of time-point measurements

Anthropometric parameters and blood pressure were measured at the following four time points: 12–14 weeks, 18–22 weeks, 29–33 weeks, and delivery. Each of these time points were converted to a time scale ranging from 0 to 1. Therefore, the initial time (i.e., before pregnancy) was considered to be time = 0, and the time of delivery was considered to be time = 1.

### Maternal anthropometrics

The height (cm) and weight (kg) of each subject were measured without heavy clothing and shoes at each time point. Data about maternal weight just before pregnancy was obtained through questionnaires. Pre-pregnancy body mass index (BMI) was categorized into the following three categories: lean or normal (16–24 kg/m^2^), overweight (25–29 kg/m^2^) and obese (30–50 kg/m^2^).

### Blood pressure assessment

The blood pressure (BP) was measured using an automated instrument (GE Healthcare Carescape™ V100 Vital Signs Monitor with DINAMAP Technology, Milwaukee, WI, USA); it was measured two consecutive times and averaged. Mean arterial pressure (MAP) was obtained according to the following formula:

$$ MAP=\frac{\left(2\times \kern0.5em  diastolic\kern0.5em  pressure\right)+ systolic\kern0.5em  pressure}{3} $$

Prior to the measurement, each of the participants was seated and asked to relax for 5–10 min. A cuff (CRITIKON Blood Pressure Cuffs®, GE Healthcare, 23–33 cm, Milwaukee, WI, USA) was placed around the non-dominant upper arm at the level of the heart, with the pressure cuff bladder midline over the brachial artery. A larger cuff (32–42 cm) was used in patients who had an upper arm that exceeded 33 cm. As enrolment in our study took place during pregnancy, we were unable to measure maternal blood pressure before pregnancy.

### Definition of normal pregnancy

To restrict enrolment to patients with normal course pregnancies, we excluded 117 (20.2 %) pregnant women who experienced any of the following events during their pregnancies: endocrine disorders, psychiatric disorders, history of bariatric surgery, secondary hypertension, gestational hypertension/preeclampsia, preterm delivery, foetal growth restriction, antihypertensive medication use, multiple gestation, and foetal death. After these exclusions, 461 women who had a normal pregnancy remained. Therefore, the study used basic inclusion criteria, including patients who were healthy or had stable chronic hypertension without known target organ involvement.

### Efforts to address potential sources of bias

Any pregnant women who were seen by our clinical investigator during the study period were considered to be potentially eligible. The pregnancy consultations were randomly scheduled by the hospital administrative staff according to the availability of the clinical investigator (L.G-M.). The patients were consecutively recruited, and the maternal anthropometric and blood pressure measurements were taken by experienced midwives who were aware of the study protocol. All of the pregnancies were supervised by the same physician (L.G-M.), but the inclusion of pregnancies in the study was determined by another researcher who coordinated the review of each clinical case (A.C.).

### Statistical analysis

Univariate analyses included the following standard statistical methods: (1) the chi-square or Fisher’s exact tests to compare frequencies from categorical variables or to study the independence between two factors; and (2) t-tests to assess the statistical significance of the difference between the means of two independent populations.

Time was considered a continuous variable, with values 0, 0.3, 0.5, 0.8 and 1, in the BMI model [respectively 0.3, 0.5, 0.8 and 1 in the systolic blood pressure (SBP), diastolic blood pressure (DBP), MAP, and BMI effect on MAP (MAP-BMI) models]. Whilst 0 and 1 were arbitrarily chosen, the other points were proportional to the observational periods in the study; namely, before pregnancy, 12–14 weeks, 18–22 weeks, 29–33 weeks, and at delivery. Hypertension was a binary variable with normotensive status as the reference category.

Linear mixed-effects (regression) models were used with the observations grouped at the individual level [19]. The random effects in the final models were identified either at the intercept alone, or at the intercept and the time coefficient. In the final models with two random effects, the best structure for the variance-covariance matrix of the random effects was shown to be that of a general positive definite matrix. Whenever the within-group error variance function had to be modelled, the assumption of independence did not seem to be compromised. For the same individual, the errors were assumed to be independent from the random effects; for different individuals, the errors were assumed to be independent. The normality assumptions for the random effects and errors distributions were assessed through graphical analysis; however, for the BMI-time model, the responses had to be log-transformed for the normality of the errors distribution to not be rejected, and the normality assumption within the remaining models was confirmed. Due to sample size constraints, interaction terms were only considered in the MAP-BMI model.

The final models were chosen based on the lowest Bayesian information criterion (BIC) or on the likelihood ratio test, as appropriate. All statistical analyses were carried out using R version 2.12.1 [20]. The significance level was set at 0.05.

## Results

Of the 461 women who were enrolled in the study, 429 were normotensive and 32 had chronic arterial hypertension.

**Fig. 1:**
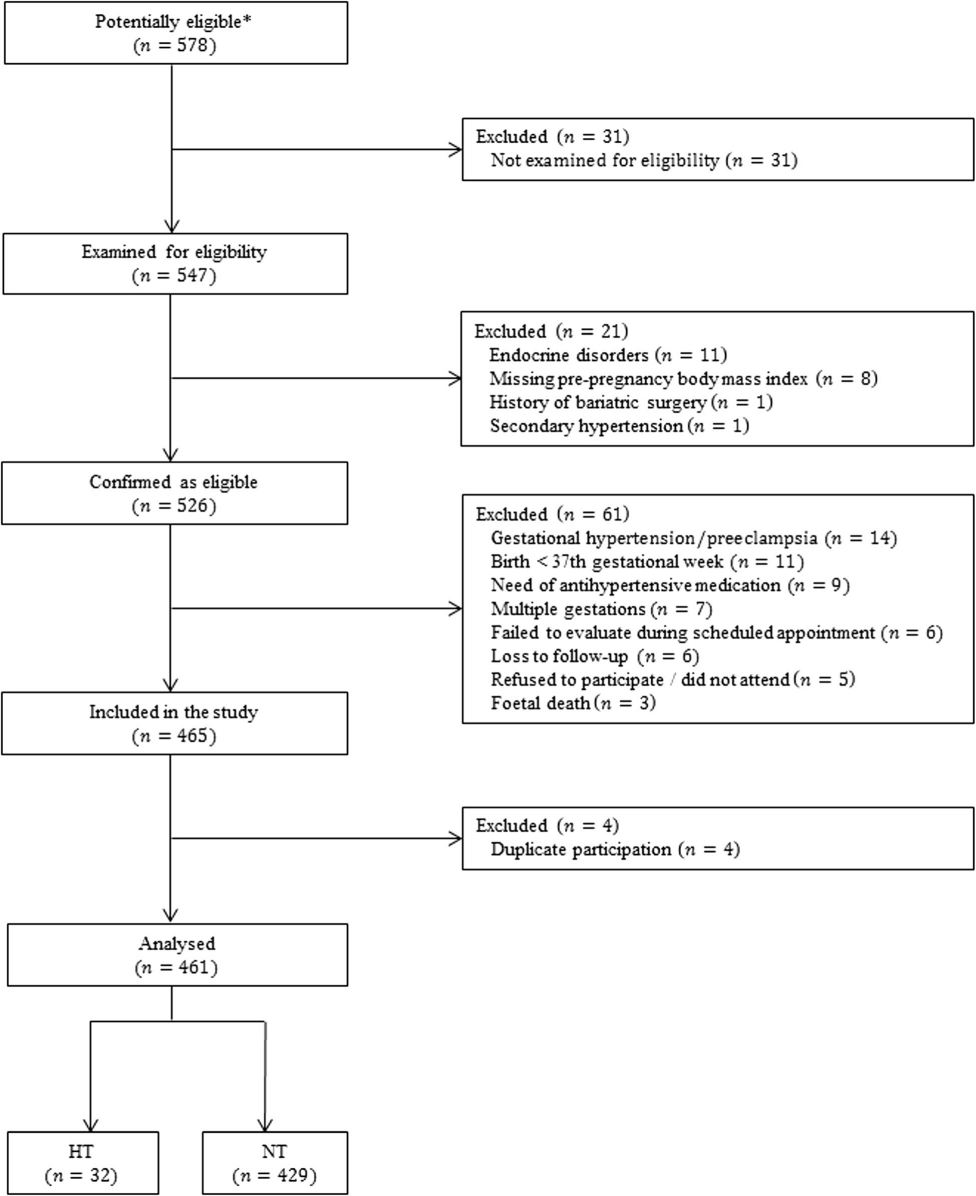
Study flowchart: number of pregnant women at each stage of the study. ^*^Any pregnant women who were seen by our clinical investigator during the study period were considered to be potentially eligible

Figure 1 shows the number of pregnant women at each stage of the study. The main characteristics and pregnancy outcomes are shown in Table 1. In 31.9 % of the cases, caesarean section was the mode of delivery; in more than 70 % of those cases, the reason for receiving a caesarean section was prior caesarean delivery, dystocia, foetal distress, or breech presentation (Table 2).

**Table 1:** Demographic characteristics and pregnancy outcomes of the 461 women included in the analysis

				Normotensive	Hypertensive		BMI (kg/m^2^)			
		*n* (%)	*p*-value^*^	*n* = 429	*n* = 32	*p*-value^**^	16–24	25–29	30–50	*p*-value^***^
Age years [years (%)]	16–24	102 (22 %)	<0.001	102 (24 %)	0	<0.001	66 (26 %)	20 (17 %)	16 (18 %)	0.047
	25–35	303 (66 %)		285 (66 %)	18 (56 %)		164 (65 %)	78 (66 %)	61 (67 %)	
	36–43	56 (12 %)		42 (10 %)	14 (44 %)		22 (9 %)	20 (17 %)	14 (15 %)	
Parity [n (%)]	0	238 (52 %)	0.485	226 (53 %)	12 (38 %)	0.140	135 (54 %)	54 (46 %)	49 (54 %)	0.335
	≥1	223 (48 %)		203 (47 %)	20 (62 %)		117 (46 %)	64 (54 %)	42 (46 %)	
Gestational age at delivery, weeks [mean (SD)]	39.22 (1.20)	-	NA	39.24 (1.17)	38.93 (1.68)	0.308	39.21 (1.18)	39.20 (1.28)	39.25 (1.22)	0.960
Foetal sex [n (%)]	Female	245 (53 %)	0.177	229 (53 %)	16 (50 %)	0.852	129 (51 %)	62 (53 %)	54 (59 %)	0.405
	Male	216 (47 %)		200 (47 %)	16 (50 %)		123 (49 %)	56 (47 %)	37 (41 %)	
Birth weight at delivery, g [mean (SD)]	3128 (334)	-	NA	3136 (329)	3007 (379)	0.070	3116 (350)	3167 (321)	3108 (301)	0.042
Apgar Score Index at 5′	<7	0	NA	0 (0 %)	0 (0 %)	<0.001	0	0	0	<0.001
	7–10	461 (100 %)		429 (100 %)	32 (100 %)		252 (100 %)	118 (100 %)	91 (100 %)	

*BMI* body mass index, *SD* standard deviation

^*^p - tested equality of population frequencies amongst the different categories of a variable

^**^p - tested homogeneity of the proportions between HT (hypertensive) and NT (normotensive)

^***^p - tested homogeneity of the proportions between normal weight, overweight and obese

**Table 2:** Indication for caesarean sections (*n* = 147) in the study sample

		Caesarean deliveries (%)^a^		
		All (%)	Normotensive (%)	Hypertensive (%)
		*n* = 147	*n* = 135^b^	*n* = 12^c^
Primary	Dystocia	29 (20)	27 (20)	2 (17)
	Non-reassuring foetal heart rate	21 (14)	19 (14)	2 (17)
	Abnormal presentation	18 (12)	17 (13)	1 (8)
	Unsuccessful trial of forceps or vacuum	14 (10)	13 (10)	1 (8)
Repeat	No VBAC attempt	40 (27)	36 (27)	4 (33)
	Failed VBAC	16 (11)	15 (11)	1 (8)
	Unsuccessful trial of forceps or vacuum	9 (6)	8 (6)	1 (8)

^a^Data are shown as absolute (relative, %) frequencies

The sums of the relative frequencies in the categories were 101 %^b^ and 99 % ^c^ due to rounding

*VBAC* vaginal birth after caesarean

Table 3 describes the maternal anthropometrics and blood pressure during the study period. BMI consistently increased over time in both the NT and HT groups. In contrast, MAP decreased during the first half of pregnancy and then increased until the moment of delivery.

**Table 3:** Description of maternal anthropometrics and blood pressure at each study period

	Period	Normotensive	Hypertensive
Weight kg, mean (SD)	Pre-pregnancy	64.09 (12.65)	75.17 (17.06)
	12–14 weeks	65.94 (13.03)	76.83 (17.67)
	18–22 weeks	69.82 (13.69)	78.69 (16.96)
	29–33weeks	75.92 (13.99)	85.03 (16.34)
	At delivery	80.96 (14.12)	92.95 (16.93)
BMI kg/m^2^, mean (SD)	Pre-pregnancy	25.10 (5.18)	28.76 (6.54)
	12–14 weeks	25.83 (5.38)	29.40 (6.79)
	18–22 weeks	27.35 (5.66)	30.12 (6.52)
	29–33weeks	29.75 (5.86)	32.56 (6.39)
	At delivery	31.73 (5.98)	35.59 (6.59)
SBP mmHg, mean (SD)	Pre-pregnancy	-	-
	12–14 weeks	119.79 (10.62)	136.22 (9.36)
	18–22 weeks	114.37 (10.28)	123.65 (9.29)
	29–33weeks	119.91 (11.03)	140.81 (8.52)
	At delivery	121.50 (11.66)	143.75 (8.84)
DBP mmHg, mean (SD)	Pre-pregnancy	-	-
	12–14 weeks	63.05 (8.15)	76.09 (6.89)
	18–22 weeks	64.09 (11.44)	72.62 (6.84)
	29–33weeks	62.93 (10.85)	78.94 (8.05)
	At delivery	66.13 (11.54)	76.88 (9.21)
MAP mmHg, mean (SD)	Pre-pregnancy	-	-
	12–14 weeks	81.96 (6.75)	96.14 (6.01)
	18–22 weeks	80.85 (8.52)	86.64 (4.45)
	29–33weeks	81.93 (8.28)	99.56 (6.45)
	At delivery	84.59 (8.82)	99.16 (6.91)

*BMI* body mass index, *SBP* systolic blood pressure, *DBP* diastolic blood pressure, *MAP* mean arterial pressure, *SD* standard deviation

### BMI model

**Fig. 2:**
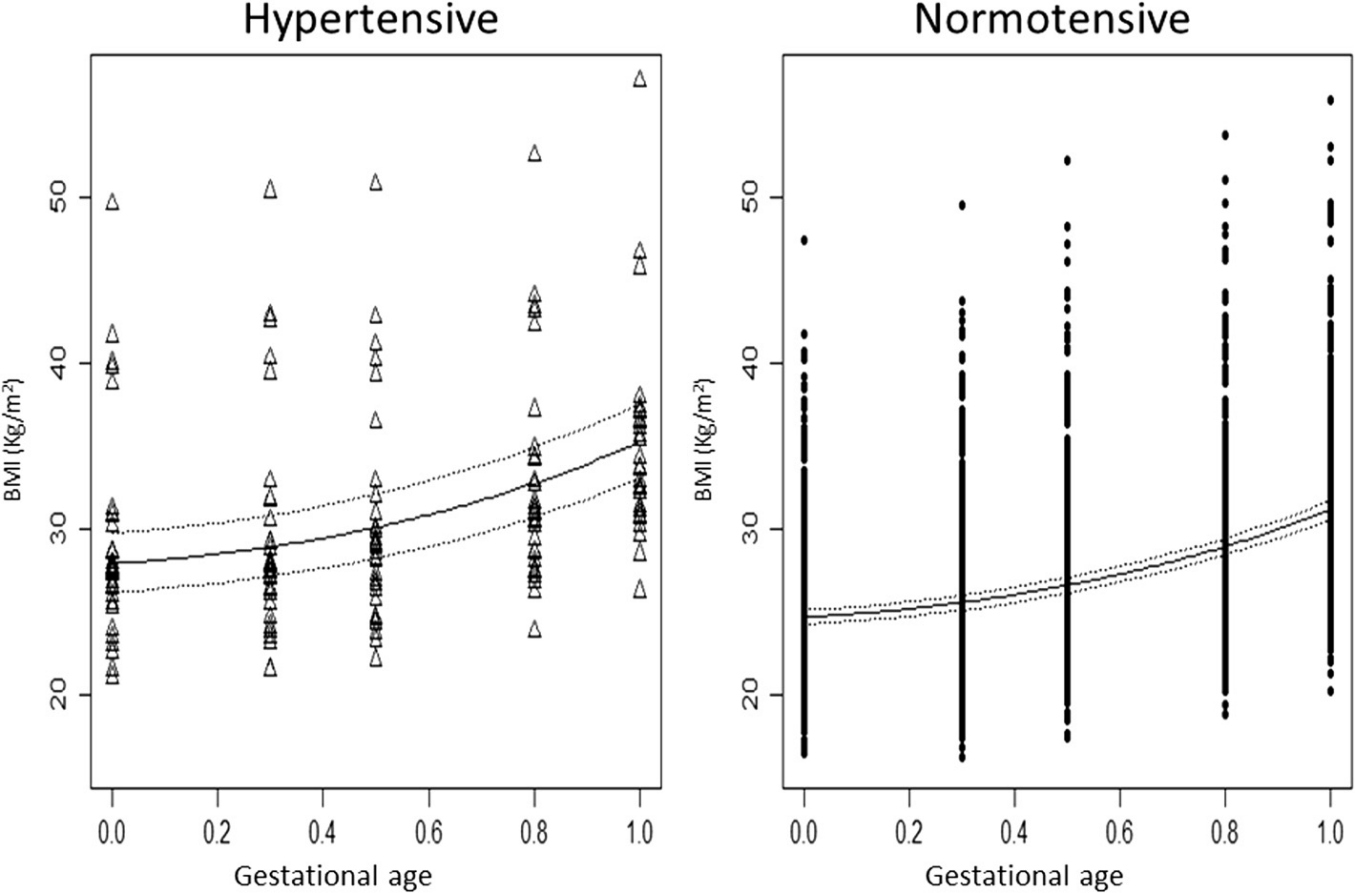
Expected body mass index (BMI) over time in the hypertensive (triangles) and normotensive (circles) women. The 95 % confidence intervals for the respective predictions are indicated (dashed lines)

The longitudinal model for BMI estimated a quadratic progression during pregnancy, with the same progression rate for both the NT and HT groups (Fig. 2).

The model for the BMI evolution during pregnancy, for any given rescaled time t and hypertensive status h (equal to 1 for HT and 0 for NT) of the i^th^ woman, was as follows:

1 $$ \log \left(BM{I}_i\right)\left(t,h\right)={\beta}_0+{b}_{0i}+{\beta}_1h+{\beta}_2t+{\beta}_3{t}^2+{\varepsilon}_i $$

where the random effect ${b}_{0i}$ was assumed to follow a normal distribution, and the within-group error terms ${\varepsilon}_i$ were assumed to follow a multivariate (5-dimensional) normal distribution with a first-order autoregressive model for their correlation structure. The corresponding estimates are presented in Table 4. The estimates for the correlation parameter (95 % CI) and the within-group standard error (95 % CI) were 0.943 (0.795, 0.985) and 0.103 (0.053, 0.198), respectively. All fixed effects are multiplicative because the response was log-transformed.

**Table 4:** Estimates for the fixed and random effects identified by the (longitudinal) mixed-effects model for body mass index (BMI), systolic blood pressure (SBP), diastolic blood pressure (DBP), mean arterial pressure (MAP), and adjusted BMI effect on MAP (MAP-BMI), in normotensive (NT) and chronic hypertensive (HT) women

	Fixed effects	Coefficient	Standard error	*p*-value	Random effects	Standard Deviation (95 % CI)
BMI	Intercept	3.206	0.009	<0.001	Intercept	0.158 (0.117, 0.212)
	HT (vs. NT)	0.124	0.034	<0.001	-	-
	Time	0.067	0.006	<0.001	-	-
	(Time)^2^	0.167	0.006	<0.001	-	-
SBP	Intercept	183.644	3.692	<0.001	Intercept	11.237 (10.058, 12.553)
	HT (vs. NT)	16.034	1.166	<0.001	-	-
	Time	−346.487	20.000	<0.001	Time	18.351 (16.601, 20.285)
	(Time)^2^	539.023	32.389	<0.001	-	-
	(Time)^3^	−253.282	16.192	<0.001	-	-
DBP	Intercept	57.967	3.905	<0.001	Intercept	7.036 (5.702, 8.683)
	HT (vs. NT)	11.954	0.974	<0.001	-	-
	Time	32.958	21.255	0.121	Time	15.327 (13.516, 17.380)
	(Time)^2^	−63.105	35.012	0.072	-	-
	(Time)^3^	38.120	17.863	0.033	-	-
MAP	Intercept	96.181	2.902	<0.001	Intercept	6.719 (5.802, 7.780)
	HT (vs. NT)	13.014	0.851	<0.001	-	-
	Time	−74.081	15.754	<0.001	Time	12.745 (11.443, 14.196)
	(Time)^2^	107.357	25.840	<0.001	-	-
	(Time)^3^	−44.409	13.116	0.001	-	-
MAP-BMI	Intercept	91.209	3.026	<0.001	Intercept	6.509 (5.604, 7.562)
	HT (vs. NT)	12.283	0.837	<0.001	-	-
	BMI	0.210	0.039	<0.001	-	-
	Time	−75.634	15.635	<0.001	Time	12.713 (11.422, 14.150)
	(Time)^2^	108.038	25.629	<0.001	-	-
	(Time)^3^	−45.221	13.004	0.001	-	-

The NT women were designated as the reference category

The predicted BMI change during pregnancy consists of two increasing parabolic branches for each hypertensive status that are significantly different from one another; for any given time, the model predicts the hypertensive population to have a mean BMI that is significantly greater than that of the normotensive population.

### SBP model

The model for SBP during pregnancy, for any given rescaled time t and hypertensive status h of the i^th^ woman, was as follows:

2 $$ SB{P}_i\left(t,h\right)=\left({\beta}_0+{b}_{0i}\right)+{\beta}_1h+\left({\beta}_2+{b}_{1i}\right)t+{\beta}_3{t}^2+{\beta}_4{t}^3+{\varepsilon}_i $$

where the random effects $\left({b}_{0i},\;{b}_{1i}\right)$ were assumed to follow a bivariate normal distribution with a general positive-definite variance-covariance matrix, and the within-group error terms ${\varepsilon}_i$ were assumed to follow a multivariate (4-dimensional) normal distribution. The estimates of the effects are presented in Table 4. The errors were found to have a variance function that is an exponent of the fitted values, with a coefficient (95 % CI) estimated to be −0.031 (−0.037, −0.025). The correlation coefficient (95 % CI) between the random effects was estimated to be −0.855 (−0.887, −0.815).

**Fig. 3:**
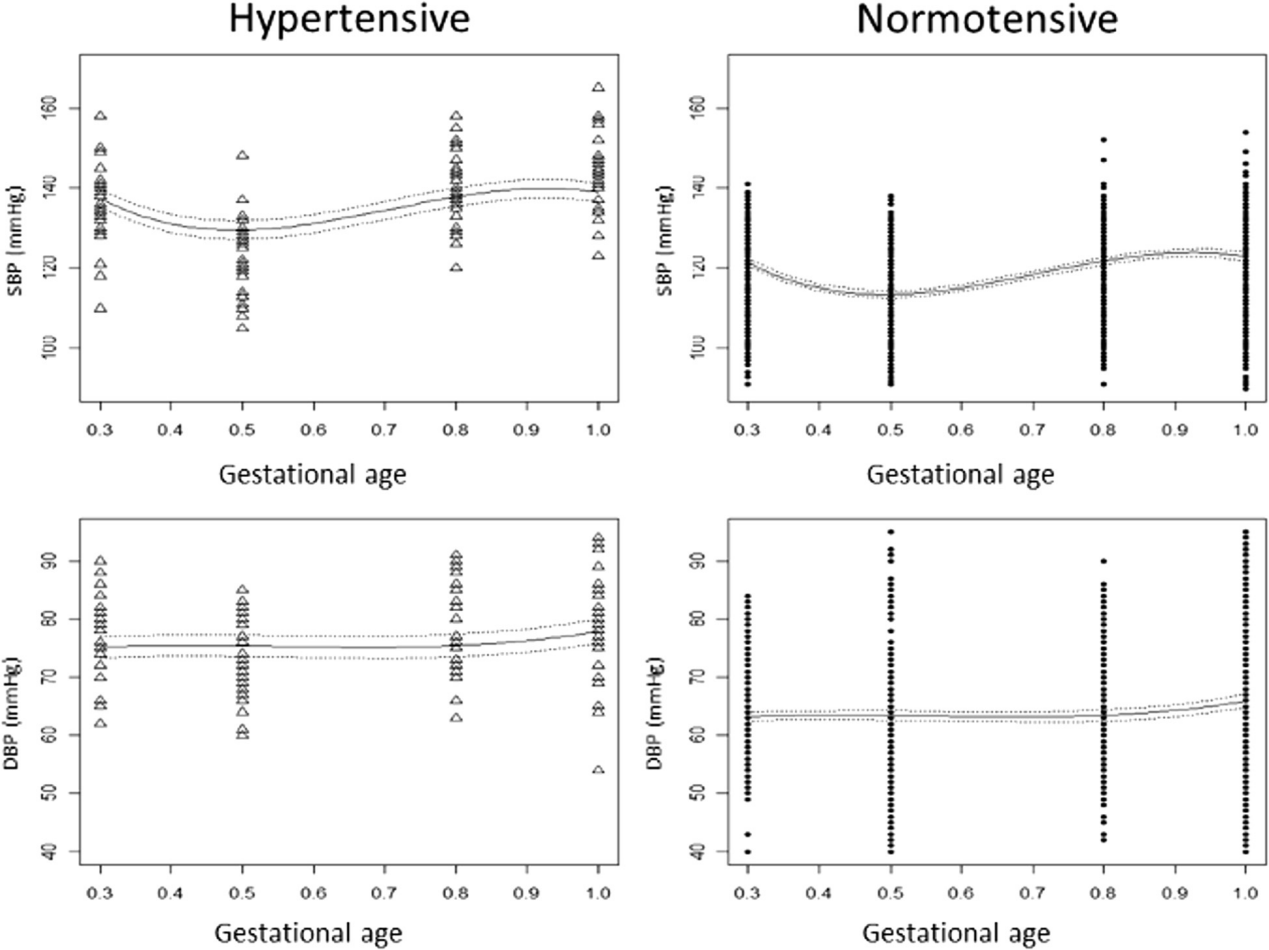
Expected systolic (SBP) and diastolic blood pressure (DBP) over time in the hypertensive and normotensive women. The 95 % confidence intervals for the respective predictions are indicated (dashed lines)

Two cubic parallel curves were obtained, one for each hypertensive status, and the SBP predictions were significantly higher for the HT group than the NT (Fig. 3). For the normotensive population, the minimum and maximum expected values were 113.5 mmHg and 123.9 mmHg, respectively, and they were attained at the (rescaled) time = 0.49 and time = 0.93, respectively. For the hypertensive population, the minimum and maximum mean values (attained at the same times) were predicted to be 129.5 mmHg and 139.9 mmHg, respectively.

### DBP model

The model for DBP during pregnancy was of a similar form to (2). The obtained estimates for its effects are presented in Table 4. The correlation coefficient (95 % CI) between the random effects was estimated to be −0.831 (−0.882, −0.760). The variance-covariance matrix of the within group errors was diagonal with a variance function that was constant for the hypertensive and normotensive groups. When the error variance within the normotensive population was standardized to 1, the estimated variance (95 % CI) of the hypertensive population was 0.691 (0.581, 0.821) times the variance prior to standardization.

Two cubic parallel curves were expected, and the DBP values were significantly higher for the HT group than the NT group (Fig. 3). For the normotensive population, the minimum and maximum mean values were predicted to be 63.21 mmHg and 66.38 mmHg, respectively, and they were attained at t = 0.64 and t = 1, respectively. For the hypertensive population, the minimum and maximum mean values (attained at the same times) were predicted to be 75.16 mmHg and 78.34 mmHg, respectively.

### MAP model

The model for MAP during pregnancy was again of a similar form to (2). Its estimates are presented in Table 4. The correlation coefficient (95 % CI) between the two random-effects was estimated to be −0.871 (−0.828, −0.772). The variance-covariance matrix of the within group errors was diagonal with a power variance function based on the fitted values; the variance function coefficient (95 % CI) was estimated to be −1.632 (−2.206, −1.058).

**Fig. 4:**
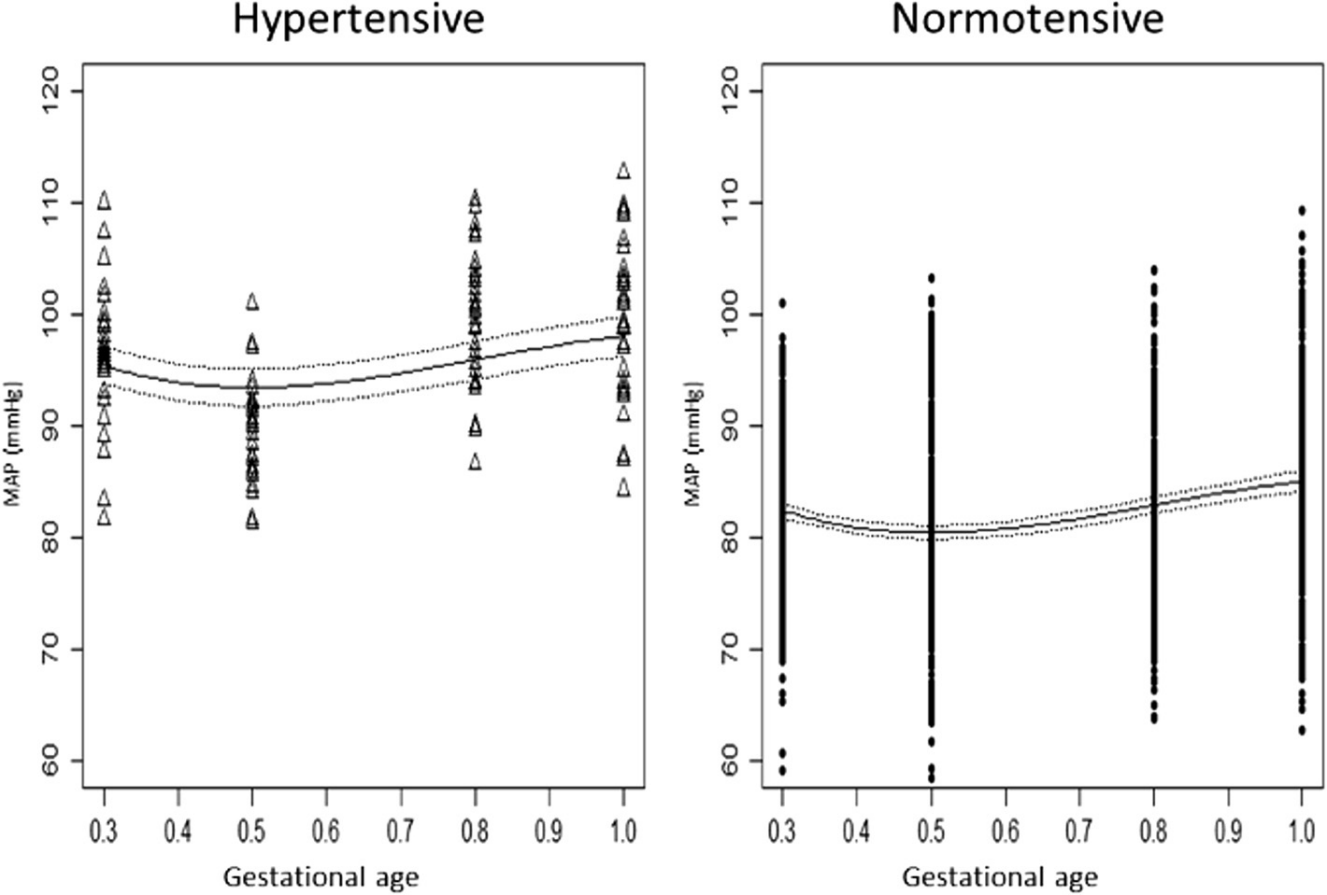
Expected mean arterial pressure (MAP) over time in the hypertensive (triangles) and normotensive (circles) women. The 95 % confidence intervals for the respective predictions are indicated (dashed lines)

For the MAP model, two cubic parallel curves, one for each hypertensive status, were predicted, and the MAP values were significantly higher in the hypertensive group than the normotensive group (Fig. 4). Between 12–14 weeks, the average MAP values were 82.42 mmHg and 95.43 mmHg in the NT and HT groups, respectively. The values decreased during weeks 18–22, reaching a minimum of 80.43 mmHg and 93.44 mmHg in the NT and HT groups, respectively. After week 22, the values increased until delivery, reaching a maximum of 85.05 mmHg and 98.06 mmHg, respectively.

### Adjusted BMI effect on MAP (MAP-BMI Model)

The effect of BMI on MAP was obtained by adjusting the previous MAP model for the time-dependent BMI variable. The estimates obtained for the model effects are presented in Table 4. The correlation coefficient (95 % CI) between the random effects was estimated to be −0.832 (−0.875, −0.776). The variance-covariance matrix of the within group errors was diagonal with a variance function that was a power of the fitted values; the variance function coefficient (95 % CI) was estimated to be −1.740 (−2.300, −1.180).

**Fig. 5:**
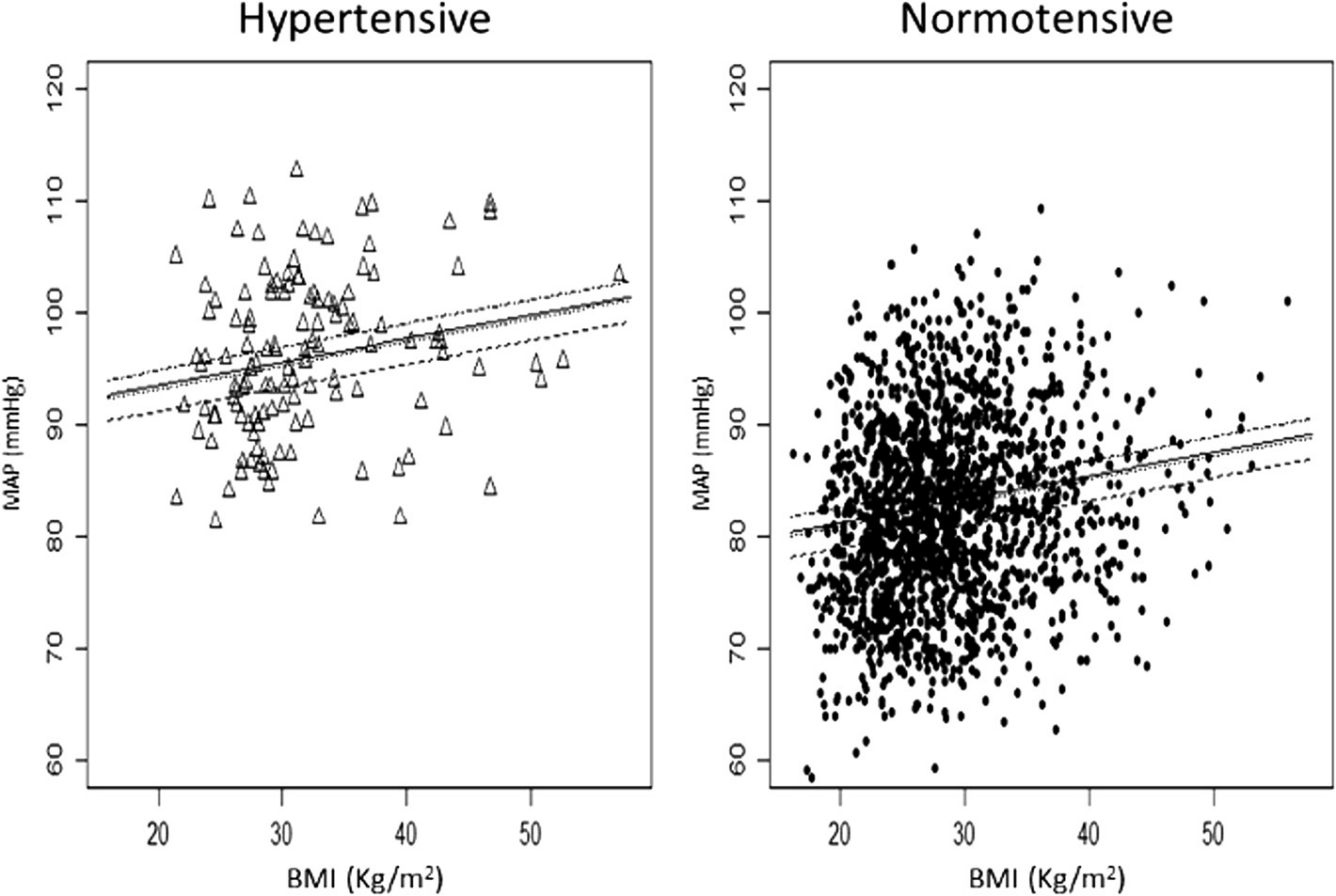
Expected effects of body mass index on mean arterial pressure, at different time points during pregnancy, in the hypertensive and normotensive pregnant women. Solid line, 12–14 weeks; dashed line, 18–22 weeks; dotted line, 29–33 weeks; dashed-dotted line, at delivery. The effect is always the same, geometrically reflected on a unique slope, regardless of the time period and hypertensive status

The interaction between hypertensive status and BMI was not statistically significant (*p* = 0.275). Adjusting for BMI led to very similar conclusions regarding the time and the hypertension effects on MAP. With respect to the BMI effect, a 1-unit increase in BMI was predicted to produce an increase of 0.21 mmHg in MAP, or equivalently, a 5-unit increase in BMI was predicted to produce an increase of approximately 1 mmHg in the population MAP values (Fig. 5). This effect was independent of time period and hypertensive status; that is, regardless of the time period and hypertensive status, the predicted BMI effect on MAP remained the same.

Parity was not statistically significant in any of the studied models (*p* = 0.443 for the BMI model; *p* = 0.712 for the SBP model; *p* = 0.471for the DBP model; and *p* = 0.729 for the MAP model).

## Discussion

Most of the normal increase in weight during pregnancy can be attributed to the foetus, breasts, and increases in extravascular fluid, and it causes inevitable demands on the hemodynamic balance of the pregnant. Consequently, blood pressure (BP) and maternal weight measurements play a central role in the adequate monitoring of pregnancy, during which profound metabolic and circulatory changes occur [13, 15, 21, 22]. These cardiovascular changes begin early and include cardiac output increase, blood volume expansion, peripheral vasodilation and blood pressure reduction. Notably, half of the cardiac output increase occurs by 8 weeks of gestation, and cardiac output increases to 30–50 % (1.8 L/min) above the typical baseline (i.e., in non-pregnant status) [23–25]. As a consequence, in early pregnancy the uterus receives 3 to 6 % of the cardiac output, whereas at 37–41 weeks it receives approximately 12 % of the cardiac output [26]. This increase is crucial for an adequate perfusion of the developing feto-placental unit [27–29].

The results from this prospective study showed that, as in normotensive gestations [30, 31], in chronic hypertensive pregnant women the shapes of the average SBP and MAP trajectories are characterized by a decrease until mid-pregnancy followed by an increase late in pregnancy. This pattern was much less noticeable for DBP, where little and almost no decreases in the average values were observed during pregnancy in the NT and HT groups, respectively. In addition, the differences in the SBP, DBP, and MAP trajectories between the NT and HT groups remained constant throughout pregnancy, and the trajectories temporally progress in a parallel fashion.

To our knowledge, this is the first study to quantify the effects of BMI on MAP in chronic hypertensive pregnant women. We observed that higher BMI values were associated with higher MAP values in all trimesters of pregnancy in both the NT group and the HT group. In addition, regardless of the time period and hypertensive status, the predicted effect of BMI on MAP remained the same; a 5-unit increase in BMI was predicted to produce an increase of approximately 1 mmHg in MAP.

Overall, our findings are in line with the robust association that has been found between increased adiposity and higher blood pressure in humans [32–34]. Particularly, previous studies have verified the blood pressure pattern that occurs during the trimesters of pregnancy in clinically healthy pregnant women [35–37] but not in a population of chronic hypertensive pregnant women.

### Statistics and methodological issues

In all of the models, the two mean curves had a narrow 95 % confidence interval. This suggests that most of the variability was captured by the random effects, which justifies their presence in the models. Moreover, the presence of random effects and the modelling of their variance-covariance structures competed with the correlation structure of the errors, which turned out to have a simple structure. This phenomenon is well-known in the statistical literature on mixed-effects models [19]. The fact that the best variance-covariance structure of the random effects was always a general positive definite matrix is in line with the values of the estimated correlation coefficients and variance estimates that were obtained in the various models.

In the DBP model, the estimate for the time coefficient was not statistically significant; however, this is irrelevant because the estimates of the higher-order terms were all significant.

No interactions between time and the hypertensive status of the women were considered. This was essentially due to the high-order polynomials (third order) that we found for the time variable combined with the sample size of the hypertensive group. Therefore, the predicted mean curves for the hypertensive population turned out to be a translation of the graphical mean values that were predicted for the normotensive population. However, this seemed to only negatively impact the fitting within the hypertensive population in the second evaluation period of the MAP model.

Within the MAP model, the residuals of the hypertensive group were larger than those of the normotensive group. This result was related to the strength of the model, particularly the cubic time progression that ignored the hypertensive status of the women, up to adding a constant. The authors predicted that the presence of interaction terms in the model would improve the model predictions for the hypertensive population. However, this method would be inadequate given the relatively low sample of hypertensive women. Nonetheless, the sample size imbalance between the NT and HT participants is in accordance with the prevalence of chronic hypertension during pregnancy [7, 8].

The principal strength of this study was its prospective longitudinal design, which allowed for the assessment of data from the first trimester onwards. In addition, our analyses are based on blood pressure and weight measurements in the clinic (routine); therefore, they reflect the patterns that occur in daily clinical practice as opposed to assessments that are made during trial conditions [15].

### Weight gain and hypertension

It is thought that weight gain causes hypertension [38, 39]. In fact, in the general population, many cohort studies indicate that being overweight is a major risk factor for the development of hypertension and that weight loss lowers blood pressure in most hypertensive patients [1, 38]. This phenomenon has been attributed to increases in weight-related sympathetic activity, which in turn result in the down-regulation of β-adrenergic receptors; this down-regulation leads to a decreased thermogenic response and, consequently, to an increased propensity for weight gain and adiposity-related insulin resistance [40]. This hypothesis has been strengthened by findings that hypertensive subjects experience a generalized decrease in β-adrenergic responsiveness, which modulates the development of obesity in hypertension [41, 42]. As a corollary, sympathetic overactivation leads to hypertension and weight gain, and the weight gain further worsens the hypertension [38, 43]. Additionally, being overweight is a cause of chronic inflammation and oxidative stress, which are involved in the pathophysiology of hypertensive disorders during pregnancy [44, 45].

Our results suggest that preventive strategies prior to conception or adequate counselling during pregnancy should be applied to prevent obesity in reproductive-age women and to promote adequate increases in BMI during pregnancy.

### Study limitations

There are several limitations to our study that should be acknowledged. First, the self-reporting of information on many covariates was generally avoided in this study, which limited the availability of detailed information about a large number of potential confounding factors, as certain adverse lifestyle-related determinants of hypertension. Second, information on maternal pre-pregnancy weight was obtained through a questionnaire; thus, it tended to be underestimated and we cannot exclude the possibility of minor misclassification. Third, the study comprised uneventful pregnancies, and thus the results cannot be extrapolated to patients with other forms of hypertensive disease during pregnancy. For the same reasons, we are not able to analyse the effect of BMI gain and the risk of gestational hypertensive disorders or any adverse pregnancy outcomes. Fourth, we were not able to measure MAP changes in relation to the distribution of maternal fat, which is more related to endothelial dysfunction, although BMI is highly correlated with visceral fat [46]. Fifth, the generalizability of our investigation is also limited because our participants were all White women. Sixth, because we did not assess pregnancies with adverse outcomes, the clinical relevance of our findings remains uncertain.

## Conclusions

This study provides evidence that in normotensive and chronic hypertensive pregnant women, MAP is strongly influenced by increases in BMI starting in the first trimester and lasting until delivery.

## Abbreviations

BIC: Bayesian information criterion
BMI: body mass index
BP: blood pressure
CHP-MJD: Centro Hospitalar do Porto – Unidade Maternidade Júlio Dinis
CI: confidence interval
DBP: diastolic blood pressure
GA: gestational age
HT: chronic arterial hypertension
MAP: mean arterial pressure
NT: normotensive
SBP: systolic blood pressure
SD: standard deviation
